# First-Hand Experience and Result with New Robot-Assisted Laser LeFort-I Osteotomy in Orthognathic Surgery: A Case Report

**DOI:** 10.3390/jpm13020287

**Published:** 2023-02-03

**Authors:** Marcel Ebeling, Mario Scheurer, Andreas Sakkas, Frank Wilde, Alexander Schramm

**Affiliations:** 1 Department of Oral and Plastic Maxillofacial Surgery, Military Hospital Ulm, Academic Hospital of the University of Ulm, Oberer Eselsberg 40, 89081 Ulm, Germany; 2 Department of Oral and Maxillofacial Surgery, University Hospital Ulm, Albert-Einstein-Allee 10, 89081 Ulm, Germany

**Keywords:** orthognatic, osteotomy, robot-assisted, robotics, LeFort-I, laser

## Abstract

Background: We report the world’s first developer-independent experience with robot-assisted laser Le Fort I osteotomy (LLFO) and drill-hole marking in orthognathic surgery. To overcome the geometric limitations of conventional rotating and piezosurgical instruments for performing osteotomies, we used the stand-alone robot-assisted laser system developed by Advanced Osteotomy Tools. The aim here was to evaluate the precision of this novel procedure in comparison to the standard procedure used in our clinic using a computer-aided design/computer-aided manufacturing (CAD/CAM) cutting guide and patient-specific implant. Methods: A linear Le-Fort-I osteotomy was digitally planned and transferred to the robot. The linear portion of the Le-Fort I osteotomy was performed autonomously by the robot under direct visual control. Accuracy was analyzed by superimposing preoperative and postoperative computed tomography images, and verified intraoperatively using prefabricated patient-specific implant. Results: The robot performed the linear osteotomy without any technical or safety issues. There was a maximum difference of 1.5 mm on average between the planned and the performed osteotomy. In the robot-assisted intraoperative drillhole marking of the maxilla, which was performed for the first time worldwide, were no measurable deviations between planning and actual positioning. Conclusion: Robotic-assisted orthognathic surgery could be a useful adjunct to conventional drills, burrs, and piezosurgical instruments for performing osteotomies. However, the time required for the actual osteotomy as well as isolated minor design aspects of the Dynamic Reference Frame (DRF), among other things, still need to be improved. Still further studies for final evaluation of safety and accuracy are also needed.

## 1. Introduction

Robotic-assisted surgery is a form of minimally invasive surgery that uses robotic technology to assist in the surgical procedure. Robot-assisted surgery was first introduced in the late 1990s [1], and it has since become increasingly popular in a variety of surgical specialties, including urology, gynecology, and general surgery. In the field of maxillofacial surgery, robotic-assisted surgery can be used to perform a variety of procedures, including jaw reconstructions, dental implant placements, and facial trauma repairs.

One of the main benefits of robotic-assisted surgery in maxillofacial surgery is its ability to improve precision and accuracy. The robotic system is able to make small, precise movements that may be difficult for a human surgeon to achieve, resulting in a higher level of accuracy and a lower risk of complications. In addition, the use of robotics allows for a greater range of motion, allowing the surgeon to access hard-to-reach areas of the face and jaw with ease [2].

In terms of safety, robotic-assisted surgery has been shown to be as safe as traditional surgery [2].

Despite the many benefits of robotic-assisted surgery, there are also some limitations to consider. One limitation is the cost of the technology, as the equipment and training required can be expensive. In addition, not all hospitals and surgical centers have the necessary equipment and trained personnel to perform robotic-assisted surgery, which may limit patient access to this form of treatment. In addition, the surgeon must be able to adapt to the unique limitations of the robotic system, such as the inability to feel tissue or provide haptic feedback [2].

Overall, robotic-assisted surgery has the potential to revolutionize the field of maxillofacial surgery by improving precision, minimizing scarring and recovery time, and providing a safer surgical experience for patients. While there are limitations to consider, the use of robotics in maxillofacial surgery is an exciting development that has the potential to greatly improve patient outcomes.

Especially in view of the virtual surgical planning, 3D printing of 3D models, incision guides, patient-specific implants, and patient-specific osteosynthesis plates, which have become the gold standard in many departments, the use of a robot can mean significant advances in patient care [3,4,5,6].

However, the accurate implementation of these digitally created preoperative plans in the operating room remains difficult; due to the quality of the imaging data, the software used for segmentation, design and planning, the experience of the engineer or surgeon performing the digital planning, the accuracy of the 3D printing or milling process, and, finally, the correct intraoperative use of the developed models, templates, implants, and osteosynthesis plates. While planning is performed virtually, the osteotomy is still performed manually and is limited by the physical properties of the osteotome. This, in turn, is subject to individual surgeon user error, and is strongly influenced by personal experience [7]. In order to digitize and automate this last step as well, the Cold Ablation Robot-Guided Laser Osteotome (CARLO^®^, AOT, Basel, Switzerland) can be used. This has already and solely been used by the developers in 14 consecutive cases for midface osteotomies. All osteotomies were within an average deviation of 0.80 mm (±0.26 mm) of the virtually preplanned location. There were no intraoperative complications or technical errors [8]. With this study, we seek to evaluate the practicality and accuracy of the CARLO^®^ for LeFort-I osteotomies in orthognathic surgery. We therefore report the world’s first developer-independent experience with robot-assisted laser Le Fort I osteotomy (LLFO) and drill-hole marking in orthognathic surgery using the CARLO^®^ device.

## 2. Materials and Methods

### 2.1. CARLO^®^ Device

The CARLO^®^ device (CARLO primo, running software version 1.1.1) comprises an Er:YAG laser mounted on a robotic arm guided by an integrated navigation system. The laser generates a beam with a focal diameter of 0.8 mm, a maximum pulse energy of 650 mJ, a maximum power of 6.5 W and a pulse length of 200 μs. It has a repetition rate of 10 Hz, a divergence angle of 81 mrad, and is classified as a class 4 laser. The target laser has a wavelength of 0.532 μm and a maximum power of 0.39 mW, which corresponds to a class 1 laser classification. The laser is operated in cold ablation mode, which allows the ablated bone surface to remain porous and biologically functional, as the temperature of the surrounding tissue never rises above 45 °C. This can be achieved by ensuring that the duration of each laser pulse is sufficiently short, the laser head is moved along the osteotomy line, and constant cooling is provided by saline sprayed from a nozzle.

A visible green laser is aligned parallel to the cutting beam of the Er:YAG laser to allow the surgeon to monitor and simulate the osteotomy path before cutting.

The robot arm (lightweight medical grade robot LBR Med, KUKA, Augsburg, Germany) to which the laser is attached has a lateral repeatability of better than 0.15 mm and an angular repeatability of better than 7 mrad. Since the robot arm is used in close proximity to the patient, its tactile sensor system is an important safety component. If the force acting on the robot exceeds a predetermined limit (e.g., in the event of a collision with a person or infrastructure), this is detected immediately, and the robot moves to its starting position or stops immediately, depending on the intensity of the collision.

The navigation system consists of the following components: 3D navigation camera on the camera trolley, LED markers in the laser head used by the navigation camera to detect the position of the laser head, and a fix patient marker mounted on the patient’s bone used by the navigation camera to detect the position of the bone.

The laser head is detected in space by means of its integrated LED markers, and its position is monitored at all times in relation to the reflectors of the patient marker, which is attached to the patient by means of a marker holder. The navigation system provides the information for the robotic arm to navigate to the osteotomy line to be cut. Disturbances, e.g., due to patient movement, are detected and compensated. In case of large deviations, the ablation laser stops and waits until the patient’s position is stable again. If the new location of the patient is within the range of the CARLO primo, the ablation laser is switched on again and the osteotomy is continued. This requires that the surgeon presses the button on the handheld all the time.

A pointer with reflective markers is used to perform patient registration and planning.

### 2.2. Pre-Operative Procedure

**Figure 1:**
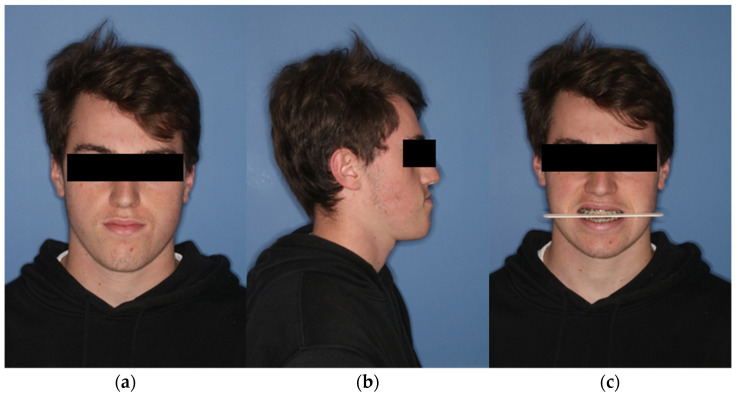
Extraoral view of the patient preoperatively. (**a**) En-face image. (**b**) Profile view from right (**c**) Frontal image showing the occlusal plane.

**Figure 2:**
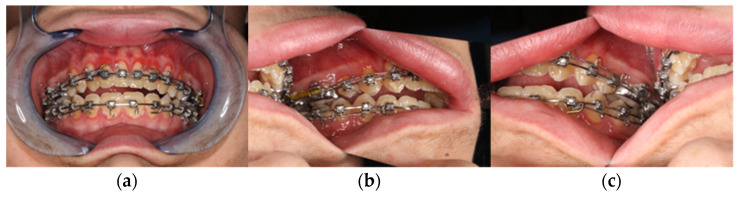
Clinical view from intraoral. (**a**) Frontal view in final bite position. (**b**) Posterior region left in occlusion. (**c**) Posterior region right in occlusion with presentation of the skeletal class III anomaly with circular crossbite and nonocclusion in the anterior region.

During the initial presentation of the 18-year-old patient with skeletal class III malocclusion, a clinical examination was performed with regard to the bite situation and the horizontal, vertical, and sagittal facial dimensions. (Figure 1 and Figure 2) In addition, the maxillary and mandibular models were scanned separately and in final occlusion using laser surface scanning.

Subsequently, a high-resolution CT of the facial skull (Somatom definition Siemens, Erlangen, Germany) and a reconstruction in 1 mm slices were performed. This then served as the basis for virtual planning in the web meeting with a medical engineer of the cooperating company.

Digital segmentation of the soft tissue, the skull, as well as the mandible and the upper skull (minus the maxilla) was performed. After completion, the existing STL files of the maxillary and mandibular models were loaded into the software and fused with the maxilla and mandible using a semi-automatic fusion algorithm. This enabled a data set with high-resolution data regarding the teeth.

This was followed by virtual planning of the Le-Fort-I osteotomy and the bilateral-sagittal split osteotomy. Then, the Surface Tessellation Language (STL) file of the maxilla and mandible in final occlusion could be imported into ProPlan CMF™, to fuse and register it to the maxilla first, and the mandible second. The mandible was then moved to the position dictated by the final occlusion. The osteotomized maxilla could then be moved in a block with the mandible to the previously determined position.

Finally, the final soft tissue was simulated using an algorithm integrated in planning software. After approval of the final result, the medical engineers manufactured the surgical cutting guides and the patient-specific implant (PSI). These finally determined the maxillary osteotomy lines and the drill hole positions. In addition, the new position of the maxilla is coded in all three dimensions in the PSI.

**Figure 3:**
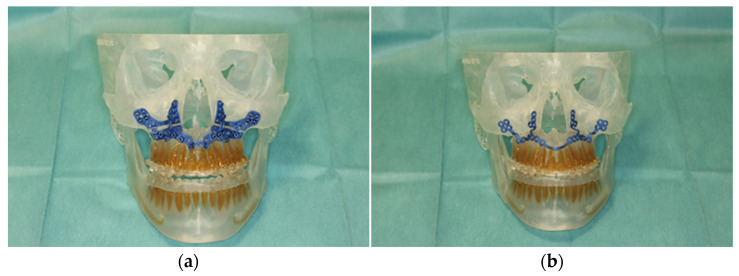
Illustration of patient-specific cutting guide (**a**) and the patient-specific implant (PSI) (**b**) on the 3D-manufactured patient skull.

After final approval of the result by the surgeon, the surgical guides and the PSI were produced using the selective laser melting technique (Figure 3).

### 2.3. Hands-on with CARLO^®^

In order to train handling of this newly applied surgical procedure using CARLO^®^, we performed a simulated operation on a 3D-printed skull of the patient in the central operating theater. For this purpose, the two surgeons, the planned surgical technical assistant, and a representative of Advanced Osteotomy Tools (AOT) were present.

**Figure 4:**
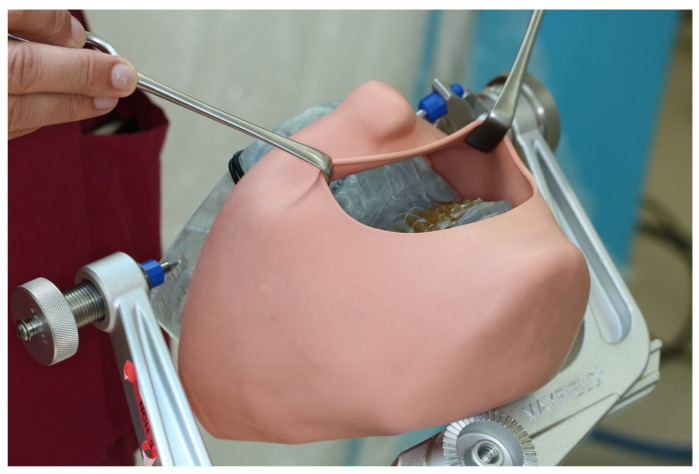
3D printed patient model fixed in the Mayfield clamp and soft tissue mask.

After instruction in the software and basic functions of CARLO^®^, the Mayfield clamp was attached to the 3D-printed skull of the patient with soft tissue dummy (Figure 4). The virtually planned Le Fort I osteotomy was exported as STL file and imported into CARLO^®^ software.

**Figure 5:**
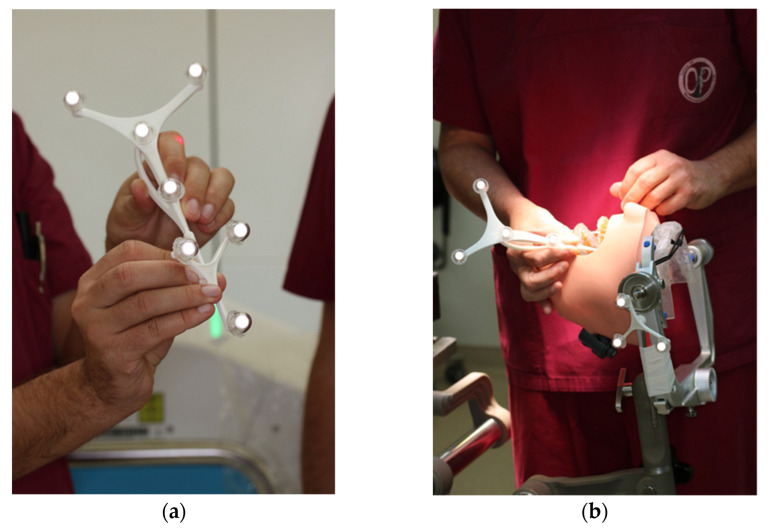
Registration process during trial surgery (**a**) Calibration of dynamic reference frame (DRF) and the navigation probe (**b**) Surface registration of at least 4 different anatomical landmarks.

The navigation probe was registered using the optical cameras and navigation software, and subsequently attached to a fixed and steady part of the Mayfield clamp. The navigation probe was positioned in direct sight of the optical cameras on the patient`s left side. Point-pair matching of at least four landmarks was used to correlate the virtual maxilla with the maxilla of the 3D skull. Dental landmarks were used for the registration process (Figure 5).

**Figure 6:**
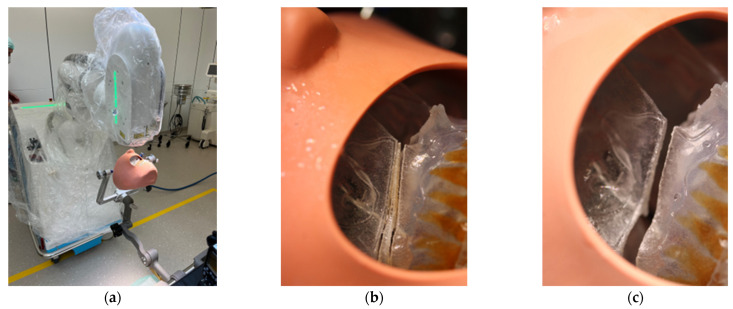
Clinical set-up of the trial surgery with 3D printed patient skull attached to the Mayfield clamp and positioning of the CARLO^®^ (**a**). Representation of the right osteotomy lines after completion of the laser osteotomy (**b**) and after removal of the “bony” wedge (**c**).

All participants wore laser protective goggles during the rehearsal. After retraction of the soft tissue dummy, the robot arm was postured in the preplanned position with the soft tissues omitted. The robot-guided laser performed the osteotomy autonomously, while still under direct control by the surgical team (Figure 6). This was accomplished without any problems, so we proceeded to mark the planned drill holes for the PSI in the 3D model using a laser beam. For this purpose, the existing data sets were overlaid with the planned PSI and also transferred to the CARLO^®^ device. Each drill hole was defined in advance, as well as the corresponding angle of incidence of the laser beam on this point. Due to the position and anatomy of the 3D model, several different angles of incidence had to be selected in order to fall approximately perpendicularly on the surface of the maxillary surface. This marking also succeeded without any problems.

## 3. Results

### 3.1. Surgical Procedure and Intraoperative Findings

The surgical procedure included our internal clinic standard of patient identification, correct anatomical patient fixation of the patient’s head in the Mayfield skull clamp, intraoral germ reduction using CHX mouthwash, intravenous administration of 3 g Unacid and 250 mg Solu-Decortin^®^ H (SDH) as swelling prophylaxis, sterile washing and draping of the patient, and subsequent team time-out.

**Figure 7:**
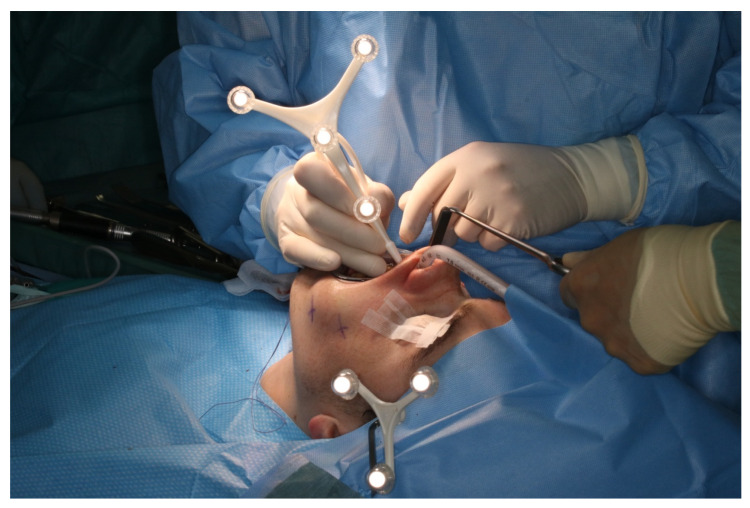
Intraoperative surface registration of at least four different anatomical landmarks with the DRF attached to the Mayfield clamp.

After vestibular incision from the right first molar to the left first molar and formation of a mucoperiosteal flap, the navigation probe was registered in accordance with the above scheme. The marker was positioned in direct sight of the optical cameras on the left side of the patient so as to not interfere with the surgeons, who were positioned to the left and right sides of the patient. A corresponding point-pair matching, according to the above-mentioned scheme, was performed (Figure 7).

**Figure 8:**
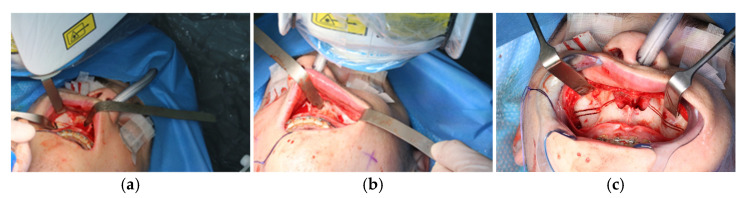
Intraoperative view of CARLO^®^ laser ablation osteotomy. (**a**) Right osteotomy with visible laser during osteotomy (green dot). (**b**) Post completion of left osteotomy. (**c**) Clinical picture after complete bilateral osteotomy and before removal of both bone wedges.

The surgeon team, nursing staff, and patient were all equipped with protective laser goggles. The laser head is automatically positioned in the operating area by the robotic arm and the osteotomy line was visualized by the green noncutting laser beam (Figure 8). The correct positioning is approved by the surgical team. Then, the laser needs to be manually turned on and controlled by a surgeon by maintaining continuous pressure on a trigger button. The robot-guided laser then performs the osteotomy autonomously while still under direct visual control by the surgical team, who are provided with real-time visualization of the cutting area on a monitor.

During the application of the laser, the oxygen saturation of the patient’s ventilation must be reduced to only 21% to prevent spontaneous ignition. This resulted in the patient’s saturation dropping below 80% due to the long laser-application time, so the laser application had to be stopped for safety reasons, allowing the patient to be ventilated again with a regular oxygen saturation. After stabilization of the saturation, the osteotomy of the maxilla could be completed.

**Figure 9:**
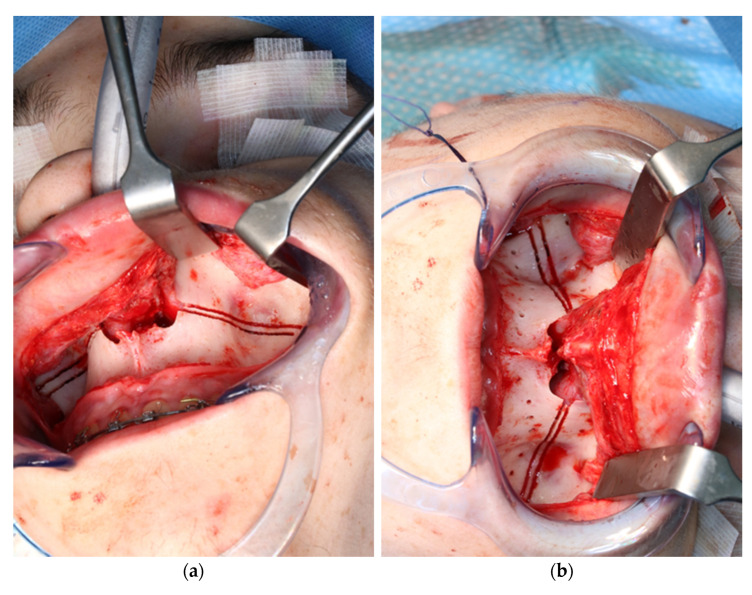
Use of the CARLO^®^ to mark the planned drill holes ((**a**), black arrows) as well as after drill holes were created using a conventional rotating handpiece (**b**).

**Figure 10:**
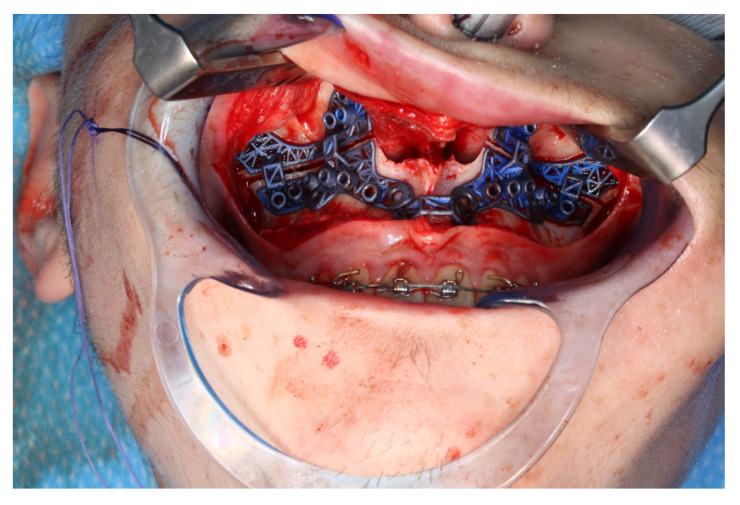
Intraoperative clinical visual control of osteotomy lines and drill holes using patient-specific cutting guide.

**Figure 11:**
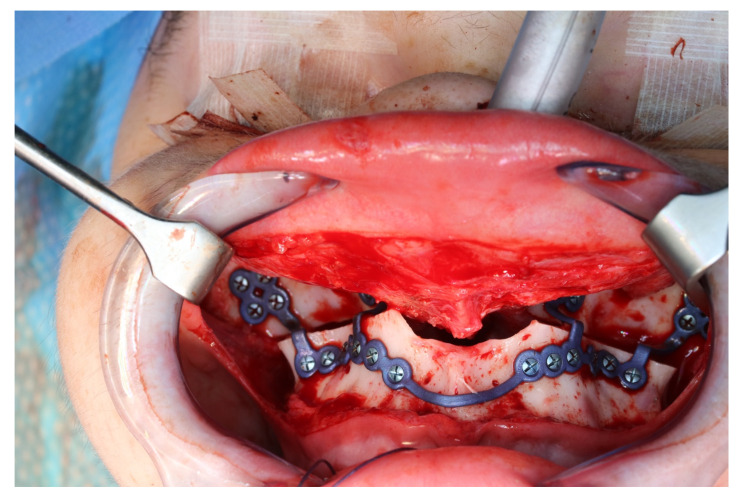
Upper jaw positioned and fixed by means of patient-specific implant.

The osteotomy of the maxilla took approximately 45 min for both sides, including spot-marking of the drilling holes (Figure 9). After the osteotomy had been performed, the CARLO^®^ device was removed, and positioning of the osteotomy lines as well as spot marking were clinically checked by superimposing the cutting guide (Figure 10). Afterwards, the osteotomy of the nasal septum, lateral nasal wall, and pterygomaxillary junction was completed using a chisel and hammer. The surgery was then completed in a conventional manner (Figure 11).

### 3.2. Postoperative Analysis

Analogous to the segmentation protocol of the preoperative CT, the segmentation of the postoperative situation was performed. The segmented objects were then imprinted into the software file, which already contained both the preoperative and the simulated postoperative situation. The registration of the segmented pre- and postoperative data sets was performed using the alignment tool of the planning software. This tool uses a global registration to align the unoperated postoperative skull base to the preoperative skull.

**Figure 12:**
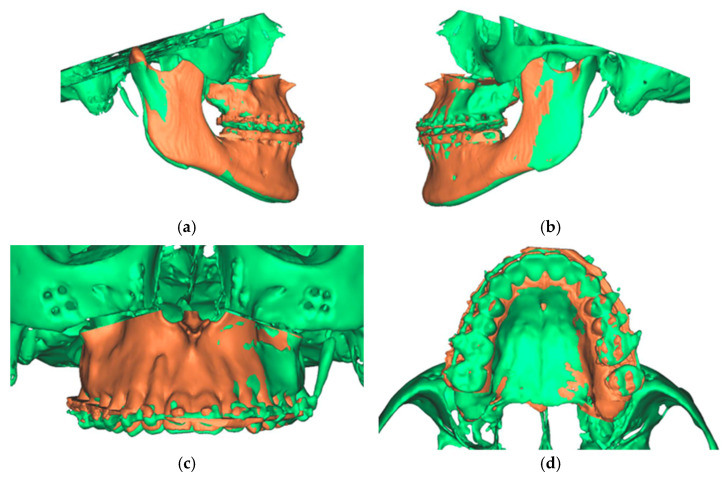
Overlay of the planned maxillary and mandibular position (green) with the postoperative position (orange). (**a**) View from the right (**b**). View from the left (**c**). View of the maxilla (**d**). View from palatal to the maxilla.

The final result was a single file containing two overlaid skulls from preoperative and postoperative situations. In addition, three different positions, preoperative, postoperative simulated, and postoperative actual, were found for the maxilla (Figure 12).

In order to compare the differences in precision and accuracy maxillary positioning with the initial planning, we defined five landmarks that could not show variability. These included the tips of the mesio-buccal cuspids of the first molars, tips of the upper canines, and the incisor point. In addition, the analysis was performed using cephalometric reference points: anterior nasal spine, the most anterior point of the tip of the anterior nasal spine in the midsagittal plane and posterior nasal spine.

For each of the five landmarks indicated on the planned maxilla object, a corresponding landmark in the preoperative position was generated. The planned position of each landmark on the maxilla was compared to the preoperative position of each corresponding landmark in the X/Y/Z-directions. The preoperative maxilla with linked landmarks was then aligned to the postoperative maxilla using the alignment tool. Once aligned, the software measured the difference and movements in X, Y, and Z axis for each of the five points (Table 1).

Analysis of our data showed that overcorrection occurred in 17% (4/24) of the measured values, with undercorrection occurring in 83% (20/24) of the measured values. The overcorrection was a maximum of 0.67 mm and a minimum of 0.06 mm with an average of 0.47 mm. A total of 75% (3/4) overcorrection occurred in the X-axis to the left, related to the incisors and canines. Only the left first molar was overcorrected by 0.06 mm in the Y axis.

The undercorrections were a maximum of 1.6 mm and a minimum of 0.04 mm with an average of 0.62 mm. All measurement points in the Z-axis were undercorrected, as well as all but one value in the Y-axis.

**Figure 13:**
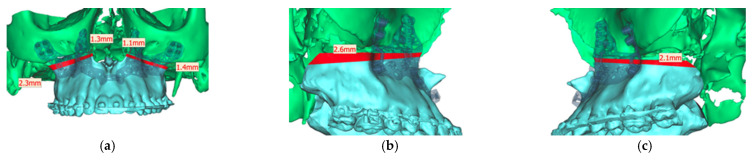
Representation of the segmented maxilla (blue) from the rest of the facial skull (green), and representation of the deviation of the osteotomy lines from the planned course in mm (red). (**a**) View from front (**b**). View from right (**c**). View from left.

Analyzing the osteotomy lines, the right side showed a divergence from a minimum of 1.3 mm to a maximum of 2.3 mm. The further the osteotomy trended laterally, the more strongly the lines diverged. A similar picture emerged on the opposite side, where the maximum deviation was also at the most lateral point of the osteotomy with 1.4 mm. At the medial beginning of the osteotomy, the deviation was 1.1 mm (Figure 13).

**Figure 14:**
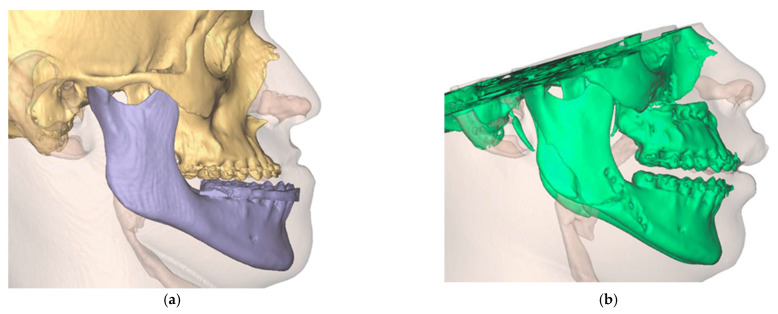
Visualization of the preoperative soft tissue profile (**a**) as well as the altered postoperative soft tissue profile (**b**).

The profile analysis showed a pre-operative SNA angle of 86.5°. An angle of 99.9° had been planned. The postoperative CT showed a deviation of −0.8° with an SNA angle of 99.1° (Figure 14).

After performing and analyzing one of the first developer-independent robot assisted Laser Le-Fort-I osteoteotomies, we summarized the advantages and disadvantages of this procedure in Table 2.

## 4. Discussion

Waferless orthognathic surgery is on the way to become the gold standard in oral and maxillofacial surgery. The fabrication of CAD/CAM cutting guides and plates enables even the less-experienced and trainees to achieve predictable results in the context of osteotomies in an acceptable time. In the context of virtual planning, there are no limits to the bone movements and the design of osteotomies respectively fragments. However, the physical properties of the bone-cutting instruments and the anatomy of the surgical region must be taken into account when manufacturing the cutting guides and plates, which relativizes the limitlessness in design and bone movement mentioned at the beginning of the paper. Therefore, it is crucial to move away from cutting guides and plates towards non-contact automated osteotomy. This could ultimately reduce the preoperative planning steps, as the STL files can be transferred directly into the CARLO^®^ software, eliminating the need to design and manufacture cutting guides and plates.

At the same time, it is important to consider that the inherent bone contact, friction, and pressure of the surgical instruments cause thermal damage to the bone and surrounding tissue, bone fragmentation, and stress in the surrounding tissues [9]. This, in turn, can lead to worsened bone healing or necrosis of individual bone parts [7,8]. Although piezoelectric instruments could minimize this risk due to their low vibration induction in the tissue and overall, less local pressure application, they could not prevent it [9,10,11,12,13].

The advantages of laser surgery are precisely the contact-free osteotomies [14,15] with the lowest geometric limitations, which allow us to create almost puzzle-like shapes. Performing a bone osteotomy using a laser offers the potential advantages of high precision, low roughness of cut surfaces, a narrow kerf, and reduced collateral damage to surrounding tissues [16,17,18]. However, even here, precision cannot be completely detached from the surgeon, so it is further dependent on the accuracy of the initial registration of the anatomical landmarks, as well as the manual skill of the surgeon.

In addition, the use of a laser theoretically allows for improved bone healing. This is due to the absence of the smear layer that occurs with rotating osteotomy instruments. This results in a channeled scaffold that preserves the trabecular ridges, which allows the passage of cells to the site of injury, therefore potentially benefiting bone healing. Several studies have shown that there is at least no difference between conventional rotary instruments and laser application [14,16,19,20,21,22].

However, we would like to point out that fixing the target marker to the hard palate of the patient means additional trauma. Additionally, we see difficulties in using it in patients with reduced mouth opening or special anatomical variations, e.g., cleft palate. We therefore recommend fixing the patient in the Mayfield clamp and fixing the target marker to a non-movable part of the clamp. This was possible without any problems in our application.

In addition, a cost- and time-effectiveness analysis should be performed to determine whether the use of the CARLO^®^ is really worthwhile for the individual clinic at significantly increased costs.

## 5. Conclusions

The authors of this paper are highly confident that robotic-assisted surgery has the potential to revolutionize maxillofacial surgery by offering a number of advantages over traditional surgical techniques, where high precision is necessary for optimal functional and esthetics. In particular, the no-longer existing geometric limitations seem to be a clear step into the future, and to justify the use not only in dysgnathia, but also in tumor surgery, trauma, and reconstructive surgery. This might spark the imagination of surgeons and help to identify and develop new approaches to surgical techniques. Of course, the time required, the increased training and education needs, as well as the costs are inhibiting factors, but so far no new technology has been perfected from the start. Le-Fort-I osteomtomy is the only approved application to date, but already BSSO is technically feasible, and the application for this is pending approval.

Overall, the use of robots in maxillofacial surgery is likely to become more widespread in the future as the technology continues to advance and improve. As a result, patients can expect to benefit from improved surgical outcomes, faster recoveries, and reduced risks of complications. It is now necessary to clarify the question of what influence different laser energies and different pulse durations have on the osteotomies; we are already planning further studies on this aspect.

## Figures and Tables

**Table 1:** Differences and movements in X, Y and Z axis post-operative (POP) of the maxilla compared to the pre-operative planning (PP) in mm.

	PP in mm	Left/ Right	PP in mm	Anterior/ Posterior	PP in mm	Up/ Down	PoP in mm		PoP in mm	Left/ Right	Anterior/ Posterior	Up/ Down	PP vs. POP	Over (+) or Under (−) correction	PP vs. POP	Over (+) or Under (−) correction	PP vs. POP	Over (+) or Under (−) correction
ANS	1.55	L	13.32	Ant	1.20	Up	1.30	L	12.70	Ant	0.58	Up	0.25	−	0.62	−	0.62	−
A	1.25	L	13.09	Ant	1.49	Up	1.21	L	12.49	Ant	0.83	Up	0.04	−	0.60	−	0.66	−
PNS	0.24	R	13.31	Ant	3.91	Up	0.25	L	12.66	Ant	2.98	Up	0.49	−	0.65	−	0.93	−
IncisorMid	0.99	L	11.94	Ant	0.97	Up	1.49	L	11.45	Ant	0.36	Up	0.50	+	0.49	−	0.61	−
CanineR	0.64	L	12.50	Ant	1.95	Up	1.31	L	11.75	Ant	0.72	Up	0.67	+	0.75	−	1.23	−
CanineL	0.67	L	11.25	Ant	0.90	Up	1.33	L	11.05	Ant	0.82	Up	0.66	+	0.20	−	0.08	−
Molar1R	0.04	R	12.86	Ant	3.26	Up	0.89	L	11.97	Ant	1.66	Up	0.93	−	0.89	−	1.60	−
Molar1L	0.01	R	11.03	Ant	1.78	Up	0.89	L	10.95	Ant	1.84	Up	0.90	−	0.08	−	0.06	+

**Table 2:** Advantages and disadvantages of using CARLO^®^ device.

Advantages	Disadvantages
No manual osteotomy by the surgeon necessary anymore, therefore less manual errors	Necessary interruption of laser osteotomy due to a drop in patient oxygen saturation with necessary oxygen reduction to 21% during the laser osteotomy
No geometric limitations	Pointer tip is not fine enough for precise marking of the referencing points
No need for cutting guides and PSI with direct digital transfer of preoperative planning to the CARLO robot	The connection of the DRF is possible in two ways, but only one works
Intraoperative adjustment of the osteotomy line quickly possible at any time by a technical assistant	Drape is rolled and not folded, and thus significantly complicates handling
Higher standardization of the OR protocol	Significantly more time-consuming than conventional procedure
Surgery by untrained personnel possible at an earlier stage of training	Partially larger surgical access necessary to ensure direct visual control of the osteotomy line
High accuracy and precision	Currently no active depth control of the laser, which makes extra protection of the tissue behind necessary
	Dependence of the accuracy of the osteotomy on the surgeon’s registration
	Fixation of the target marker on the hard palate causes additional trauma
	The water-cooling system creates a spray that contaminates the whole operating field and surgical team
	Massively high acquisition costs
	Additional, non-sterile personnel required to control and guide the robot

## Data Availability

Data is unavailable due to privacy restrictions.
